# Isolation Associated Aggression – A Consequence of Recovery from Defeat in a Territorial Animal

**DOI:** 10.1371/journal.pone.0074965

**Published:** 2013-09-06

**Authors:** Paul A. Stevenson, Jan Rillich

**Affiliations:** 1 Institute for Biology, Leipzig University, Leipzig, Germany; 2 Institute for Neurobiology, Free University of Berlin, Berlin, Germany; USDA-Agricultural Research Service, United States of America

## Abstract

Population density has profound influences on the physiology and behaviour of many animal species. Social isolation is generally reported to lead to increased aggressiveness, while grouping lowers it. We evaluated the effects of varying degrees of isolation and grouping on aggression in a territorial insect, the Mediterranean field cricket, *Gryllus* *bimaculatus* . Substantiating early observations, we show that dyadic contests between weight-matched, adult male crickets taken from groups rarely escalate beyond threat displays, whereas interactions between pairs of previously isolated crickets typically escalate to physical fights lasting several seconds. No significant differences were found between 1, 2 and 6-day isolates, or between individuals grouped for a few hours or lifelong. Unexpectedly, crickets grouped in immediate proximity within individual mesh cages that precluded fighting while permitting visual, olfactory and mechanical, antennal contact, were as aggressive as free isolates. This suggests that reduced aggression of grouped animals may be an acquired result of fighting. Supporting this notion, isolated crickets initially engage in vigorous fights when first grouped, but fighting intensity and duration rapidly decline to the level of life-long grouped crickets within only 10 min. Furthermore, grouped crickets become as aggressive as life-long isolates after only 3 hours of isolation, and on the same time course required for crickets to regain their aggressiveness after social defeat. We conclude that the reduced aggressiveness of grouped crickets is a manifestation of the loser effect resulting from social subjugation, while isolation allows recovery to a state of heightened aggressiveness, which in crickets can be considered as the default condition. Given the widespread occurrence of the loser effect in the Animal Kingdom, many effects generally attributed to social isolation are likely to be a consequence of recovery from social subjugation.

## Introduction

Social isolation results in dramatic behavioural and physiological changes in a wide variety of animal species from insects to man [1]. In mammals, the so-called isolation syndrome serves as a model for psychoneurosis and is characterised by changes in corticosterone levels, neurotransmitter systems, metabolism, growth and behaviour [2]. A similar isolation syndrome is evident in insects [3], for which social isolation has wide spread and dramatic effects on physiology, behaviour and even appearance of the animals [4,5].

With respect to aggression, numerous studies on vertebrates have noted that individuals reared in isolation have higher aggression, whereas aggression is depressed in individuals reared together [6,7]. Although isolation is often viewed as a pathological condition that can lead to increased aggression [8], experimental findings and opinions on this vary due to differences in defining aggression, methodology and whether the animals investigated are more social or territorial [6,9]. Social isolation is also generally reported to increase aggressive behaviour in insects such as crickets [10–12], solitary wasps [13] and fruit flies [14,15]. A wide variety of ultimate causes for isolation associated aggression have been discussed and are still currently debated [8,9,16] and include accumulated aggressive motivation [17], higher stress or arousal [18], hyperactivity due to increased sensitivity to environmental stimuli following sensory deprivation in isolation [19], the establishment of resident dominance in the enclosure which mimics the acquisition of a territory [20], recovery from pre-isolation habituation to agonistic assaults [21,22], removal from social inhibition of aggression [23] or forgetting prior agonistic experience in isolation [24].

The proximate mechanisms underlying isolation-associated aggression are mostly obscure. Isolation, crowding and stressful conditions are frequently associated with changes in neurochemical systems, such as neurosteroids, amino acids and biogenic amines in mice [25–29] and biogenic amines in insects [12,30–32]. Changes in the functioning of such neuromodulators are generally thought to play a key role in orchestrating social behaviour both in insects [33] and vertebrates [34]. In insects, biogenic amines in particular are renown for their controlling influences on aggression [35–37]. We have previously shown that experiences as diverse as flying [38], residency [39] and winning an agonistic encounter [40] each lead to increased aggressiveness in field crickets via the action of the biogenic amine octopamine, the invertebrate counterpart to noradrenaline. It is hence temping to speculate, that isolation-associated aggressiveness in crickets may reflect changes in the operation of the octopaminergic system, for example as suggested for dopamine and isolation induced stress and aggression in mice [28].

In this paper, however, we address an alternative, and largely neglected possibility that socially isolated crickets become hyper-aggressive due to recovery from earlier social subjugation while grouped. As in many animals [7], social defeat in crickets is followed by a period of suppressed aggressiveness that can last several hours [10,11,41–43]. The data presented here show that recovery from the loser effect can fully account for isolation-associated aggression in crickets.

## Materials and Methods

### Experimental animals and groups

All experimental animals were mature 2-3 week old adult male Mediterranean field crickets (Gryllus *bimaculatus* de Geer) obtained from a breeding colony kept under constant standard conditions at Leipzig University (22–24°C, relative humidity 40–60%, 12h: 12h light: dark regime daily feeding on bran and fresh apples and carrots, and moistened daily with a water spray). The animals were maintained in mixed sex groups from the day of hatching in clear plastic terraria (width, length, height: 20x36x28 cm) with sand on the floor and containing egg cartons for shelter. Males for the experiments were separated from females on the day of the adult moult and kept as groups of 20-30 animals per cage (average density: 350 males/m^2^). The influences of social isolation and crowding on aggression was evaluated from the following groups of experimental animals:

#### Crowded colony

These males were taken directly from the colony, maintained under the conditions described above.

#### Grouped

These animals were taken from the crowded colony and then kept as groups of 10, 20 or 30 males per terrarium (sized as above), with sand and ample food on the floor, but without egg cartons as shelter, for at least 5 days prior to experimentation. This gave a more restricted distribution of animals in a two dimensional plane that allowed us to view all animals continually. These animals were tested after different periods of grouping (given in results). In one series of experiments groups of 20 males were housed together with 10 adult, mature, virgin females.

#### Isolated

These animals were taken from the crowded colony and each maintained in an individual glass jar (diameter 7cm, height 10 cm) that had a perforated plastic lid and the walls covered with black paper. The base was covered with sand as substrate and ample food provided. The animals were tested after different periods of isolation (given in results). In one series of experiments each isolated male was housed (minimum 24 hours) together with a single mature, adult, virgin female with which it freely copulated.

#### Caged isolated

These crickets were taken from the crowded colony and placed in individual cages of sufficient size to just accommodate the occupant (width, length, height: 4x4x3cm). The four sides were fashioned from thin (0.5 mm) aluminium sheeting with punched rectangular holes (8x8mm; frames 3.5 mm) through which the animals could extent their antennae, but not escape. The base of each cage was constructed from opaque grey plastic and the top from clear acrylic plastic with a central hole (8 mm diameter). Ample food was added to each cage, and the caged placed in individual glass jars (as above). The animals were tested 18-24 hours after being caged and isolated.

#### Caged grouped

These animals were taken from the crowded colony and housed in the individual cages described above, and a total of 32 (4 rows of 8) placed adjacent to each other in a standard sized terrarium. These animals thus had visual and olfactory contact, and frequent contact with each other via their antennae, but could not fight. Only animals with neighbouring cages on all four sides (12 /terrarium) were tested, 18-24 hours after being caged and grouped.

#### Re-grouped

These crickets were first isolated from the colony for at least 24 h and then 10 animals re-grouped in the standard sized terrarium. Each individual was initially placed under a plastic cup, and the cups removed all at once via an attaching cord. A video camera (Panasonic VW-CP5500) positioned above the terrarium recorded the events from grouping onwards. The sequences were subsequently examined to evaluate all aggressive interactions occurring within 5 successive 2 min periods (10 min in all). Data from 5 different experiments, each with different animals, were pooled (50 animals in total).

#### Losers

These animals were taken from a crowded colony, kept isolated in individual glass jars for 18-24 hours and pairs of similarly weighted animals matched against each other in fights (details below) that resulted in clear winners and losers. Pairs of weight-matched losers from these fights were then matched against each other at different times after the first defeat.

All treatments of the experimental animals complied with the Principles of Laboratory Animal Care and the German Law on the Protection of Animals (Deutsches Tierschutzgesetz). Different crickets were used for each experiment performed. The data presented here is based on observations of the behaviour of 1010 individual male crickets (1170 with supplementary data).

### Evaluation of aggression

In the majority of experiments, we evaluated the interactions between pairs of similarly weighted (< 5% difference) crickets of the same experimental group. In one experimental series individual males of selected experimental groups were matched against similarly weighted isolated mature adult males that had previously been induced to fly for 3 minutes by suspending them from a small holder glued to the pronotum in the warmed air stream of a commercial grade hair dryer. Since flying greatly enhances the expression of aggression in crickets [38,42], experimental animals could thus be tested against highly aggressive, near standard, opponents. In all experiments aggression was evaluated maximally 5 min after removing the animals from their respective containers. For each contest, two males were placed at opposite ends of a small, Perspex-glass fighting arena (16 x 9 x 7 cm) having a sand-covered floor and divided by a sliding door. On raising the door the crickets contact each other within seconds. The ensuing agonistic behaviour follows an escalating sequence of stereotyped motor performances [38,44], which do not differ significantly to fights that occur in the field as part of their normal behavioural repertoire [10,45].

The intensity of observed aggressive interactions were scored on a scale of 0-6 [42,44] denoting the level to which a fight escalates before the winner is established by the retreat of one contestant: Level 0: mutual avoidance without aggression. Level 1: one cricket attacks, the other retreats. Level 2: antennal fencing. Level 3: mandible spreading by one cricket. Level 4: mandible spreading by both crickets. Level 5: mandible engagement. Level 6: grappling, an all-out fight involving repeatedly engagements and biting. The interactions can be concluded at any of the levels by one opponent retreating, whereby the winners generally generated the characteristic rival song, and body jerking movements (these two later behaviours occurred in 45% and 95% respectively of all clearly aggressive interactions between males, irrespective of the test group and with no differences between the groups). Fight duration, from first contact until conclusion, was measured to the nearest second with a stopwatch; the duration of any pauses that occasionally occurred when the animals lost contact were deducted. All experiments were performed during daylight hours at room temperature under laboratory conditions, but avoiding times when we have noted that aggression tends to be depressed (just after midday and on generally dreary days, see 44,46). To minimise differences in groups due to daily variations in performance, we took the precaution of testing an equal number of pairs of crickets from each different group during each daily experimental session, and accumulated sufficient numbers from multiple daily sessions, whereby the sequence of tested groups was random for each day.

### Statistical analysis

All statistical tests were performed using standard commercial software (Prism 5, GraphPad Software Inc, La Jolla, CA, USA) running on a Power Macintosh computer (Apple Computers, Cupertino, CA, USA). The median and the interquartile range (I.Q.R.) were calculated for non-parametric data sets. The Mann–Whitney U-test was used to test for significant differences in the distributions between 2 (unpaired) data sets (non-parametrical tests were performed on duration since the data sets failed D’Agostino and Pearson omnibus normality tests, even after log transformations). The Kruskal-Wallis was used to compare three or more unmatched groups, and the Chi-squared test for differences in win frequencies. Due to greater chance of committing type II than type I errors following Bonferroni correction of alpha [47,48], we avoided applying it routinely, and instead specifically indicate error probabilities that failed significance after Bonferroni correction in the few instances where this occurred.

## Results

### Effects of grouping and isolation on aggression

**Figure 1:**
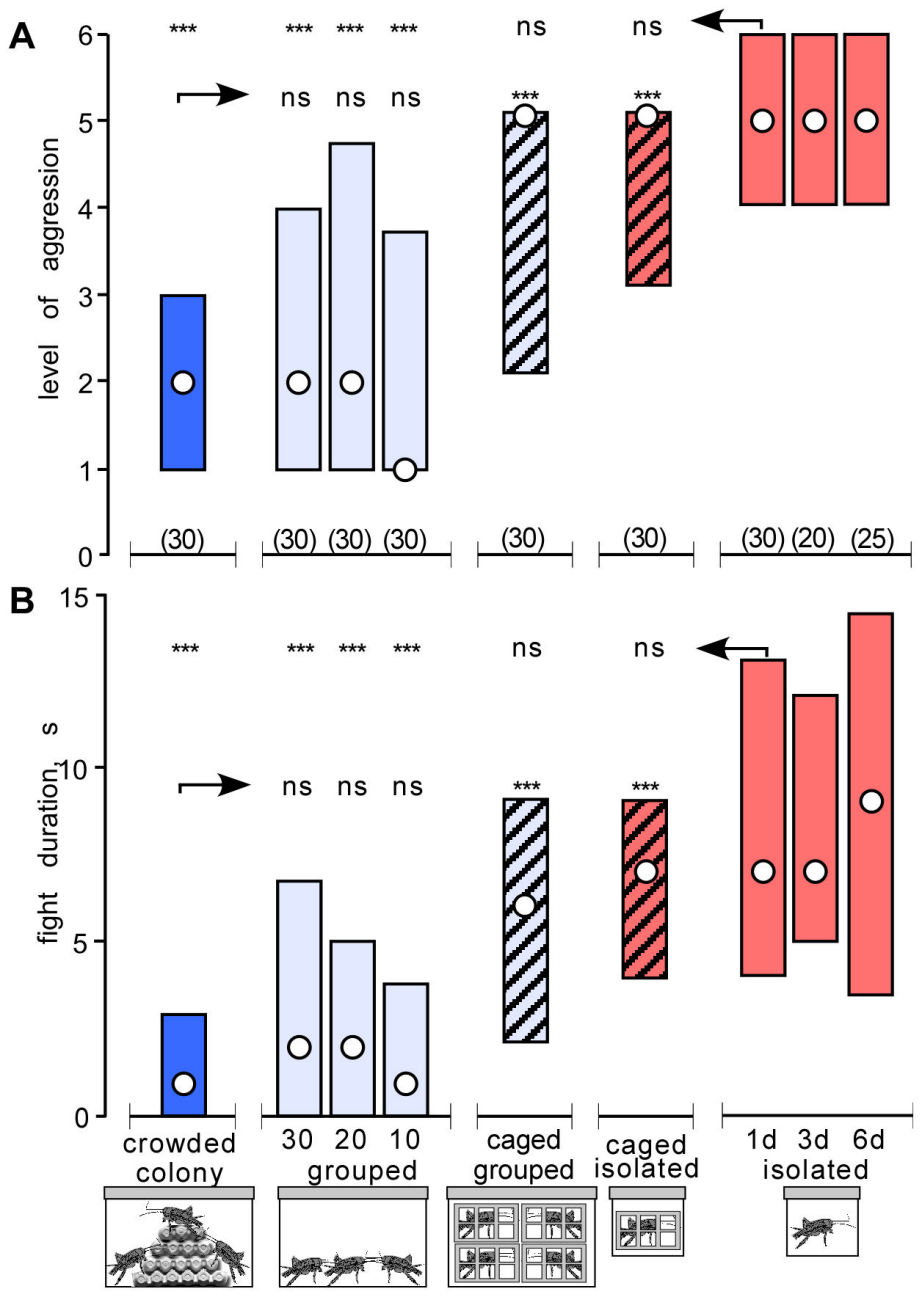
Effects of crowding and isolation on cricket aggression. (A, B) Bar graphs giving the level and duration respectively (circles: medians, bars: interquartile range) of aggression for encounters between pairs of male, weight matched crickets taken from the same test groups (from left to right): crowded colony (dark blue), grouped (light blue: groups of 30, 20, and 10 individuals as indicated), caged-grouped (light blue, hatched), caged-isolated (red, hatched), isolated (red: for 1, 3 and 6 days as indicated). Numbers in parentheses above the x-axis in A give the number of pairs or crickets for each group. Significant differences between groups are indicated (Mann–Whitney U-test, * p < 0.05, ** p < 0.01, *** p < 0.001, ns not significant).

In accord with earlier observations (e.g. [10,42]), crickets matched against each other immediately after taking them from our crowded colony exhibited a relatively low level of aggressiveness (Figure 1). Their interactions were usually concluded with antennal fencing (median level 2, interquartile range, IQR, 1-3) within only a second (median 1, IQR 0-3) and rarely escalated beyond the stage of mandible spreading (level 3). In comparison, crickets isolated for 1 day from the colony nearly always exhibited physical fighting (median level 5, IQR 4-6) in interactions lasting several seconds (median 7, IQR 4-13; level and duration both significantly different to crowded; U tests p< 0.001). Longer periods of isolation (e.g. 3 and 6 days, Figure 1) did not appear to result in any further change in aggressiveness.

Crickets taken from the crowded colony and maintained in controlled groups of 10, 20 and 30 individuals in terraria that offered no shelter or possibility of climbing above ground level, exhibited aggressive behaviour equivalent to that shown by crickets from the crowded colony, with no indication of any change resulting from the differences in population density (differences between median levels and median durations not significant for all groups; Figure 1). Contrasting this, caged-grouped crickets, i.e. grouped but separated by individual cages that permitted visual, olfactory and mechanical antennal contact, were significantly more aggressive than crowded colony crickets (level and duration both significantly different; U tests p< 0.001). In fact, caged grouped crickets were equally as aggressive as cage isolated caged crickets (U tests: level p=0.35, duration p=0.41) and fully isolated crickets (level: U tests: level p=0.08, duration p=0.25).

In a complementary series of experiments to those depicted in Figure 1, we evaluated the aggressiveness of grouped, caged grouped, caged isolated and isolated crickets when matched against similarly aged and weighted males that were previously flown in order to make them highly aggressive (Figure S1). These interactions were invariably initiated by the flown animals, which were first to attack or spread their mandibles. A comparison between test groups again revealed that caged isolated and caged grouped crickets were as aggressive as 1 day isolated males with respect to the level and duration of aggression (U tests, p>0.05), but significantly more aggressive than grouped crickets (U tests: level p=0.0042 and 0.0011 respectively; durations p< 0.001). These differences in the aggressiveness of the groups were in part also reflected in the probability of winning. While grouped males won only 5% of interactions against flown isolates, caged grouped crickets won 20% of such fights, the caged isolates 25% and 1 day isolates 30% (only latter significantly different to grouped: p=0.0375; CHI square 4.329).

### Influence of females

**Figure 2:**
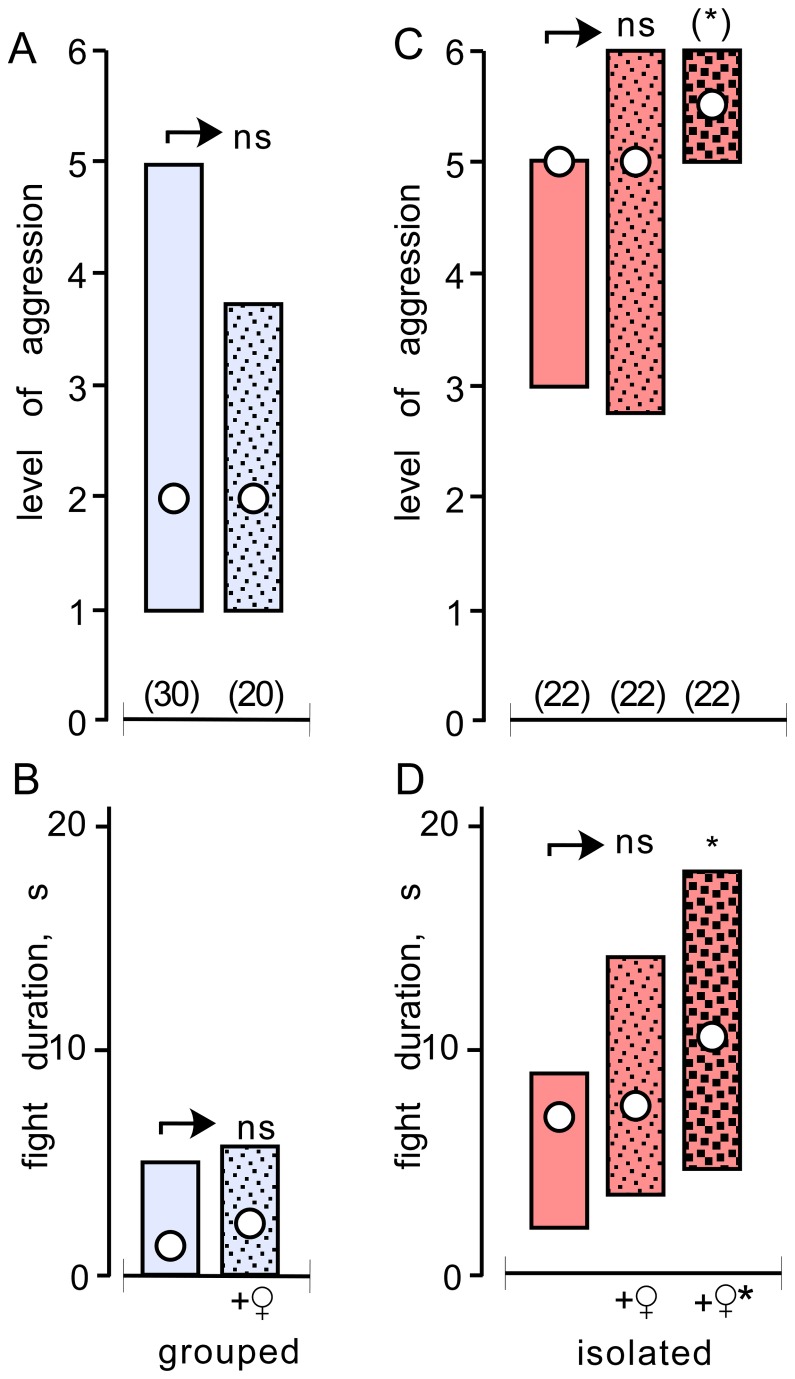
The influence of females. (A, B) Bar graphs comparing the level of aggression and fight duration respectively (circles: medians) exhibited by pairs of weight matched grouped crickets (light blue bars, compare also with Figure 1) compared to pairs of weight matched crickets taken from groups of 20 males together with 10 mature, adult, virgin females (stippled light blue bars). (C, D) Bar graphs comparing the level and duration of aggression respectively (circles: medians) exhibited by pairs of weight matched, isolated male crickets (light red bars, compare also with Figure 1) compared to pairs of weight matched isolated males that were each housed with an individual mature, adult, virgin female (stippled light red bars). The third bar in series (light red, darkly stippled) depicts fights between isolated males housed with females, as previous, whereby the females were also present in the fighting arena. Significant differences between groups are indicated (Mann–Whitney U-test, * p < 0.05, ** p < 0.01, *** p < 0.001, ns not significant). Differences that do no survive Bonferroni correction for alpha are placed in parentheses (*).

To discriminate effects of isolation and crowding from influences due to the absence or presence of females, we evaluated the aggressiveness of males grouped together with 20 other males and 10 adult virgin females, as well as of isolated males housed together with an individual virgin female. As shown in Figure 2A and B, the aggression exhibited by males grouped with females was not significantly different to that exhibited by males kept in groups of males without females (level: U test p= 0.660, duration: U tests p=0.527; fights in absence of females). Likewise, isolated males housed together with a single virgin female were equally aggressive as males isolated without a female (Figure 2C and D, level: U test p= 0.665, duration: U tests p=0.311). However, isolated males housed with a single female tended to fight more aggressively when two females were also present in the fight arena during the contest (U tests: level p=0.030 – not significant after Bonferroni alpha correction, duration p=0.0243; Figure 2 C and D heavily stippled red bars).

### Acquisition of the crowding effect

**Figure 3:**
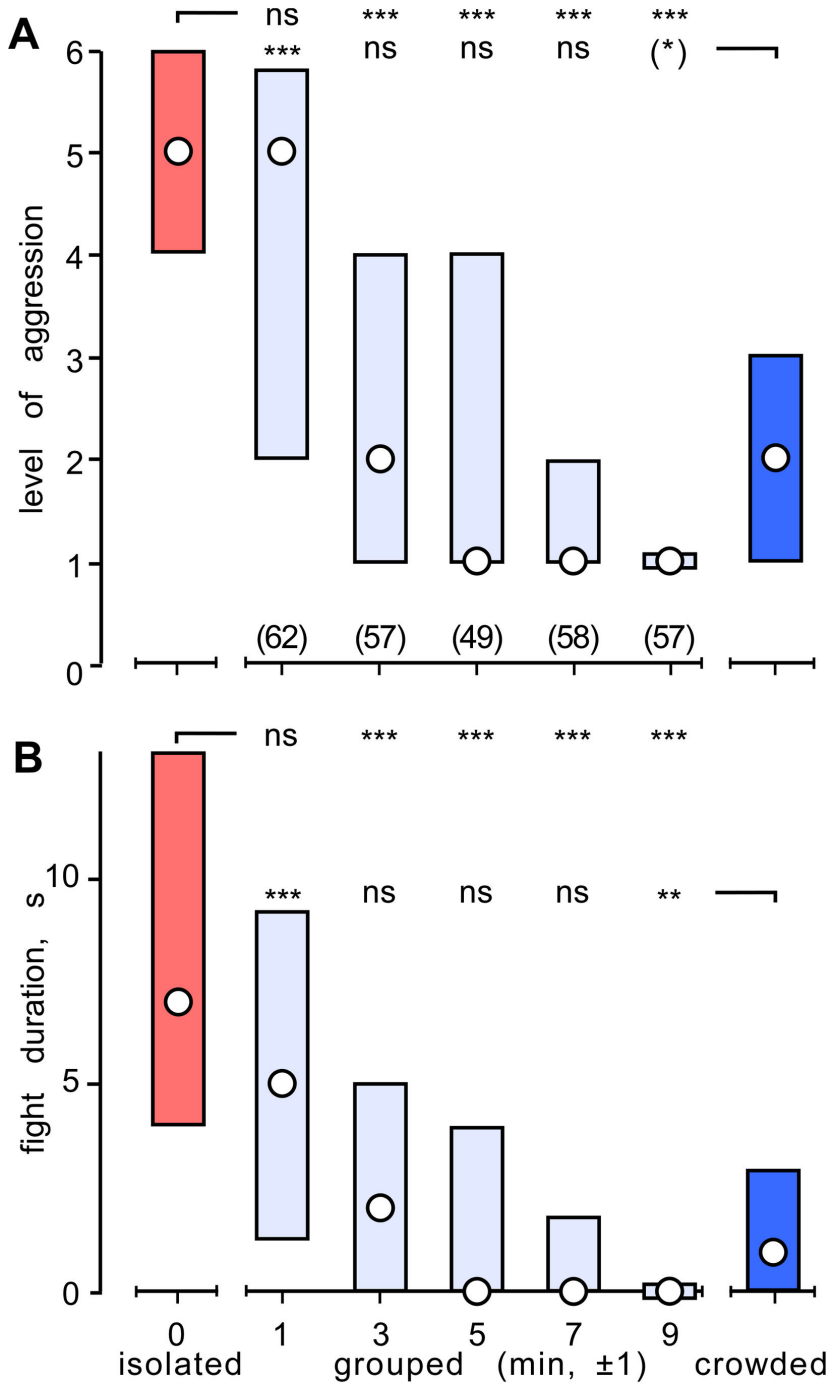
Acquisition of the crowding effect. (A, B) Bar graphs giving the level and duration respectively (circles: medians, bars: interquartile range) of aggression for encounters between pairs of male, weight matched, initially isolated crickets after being re-grouped for various lengths of time (pale blue bars). Each bar gives data accumulated from 5 separate observations, each with 10 different re-grouped crickets, for which all interactions occurring within the observation period were evaluated (n, given above the x-axis in A). Data for isolated crickets (red bar) and crickets taken from our crowded colony (blue bar) are included for comparison. Significant differences between groups are indicated (Mann–Whitney U-test, * p < 0.05, ** p < 0.01, *** p < 0.001, ns not significant). ). Differences that do no survive Bonferroni correction for alpha are placed in parentheses (*).

Our findings so far suggest that the lower aggressiveness of crowded crickets may be an acquired result of fighting. To test this, initially isolated crickets were re-grouped by setting them free in a terrarium (10 individuals per terrarium, 5 terraria in all) and their interactions observed for 10 min (Figure 3). During the course of the first observation period (0-2 min) we observed a total of 62 dyadic interactions for all 5 trials. The level and duration of aggression for these interactions was not significantly different to that for isolated crickets (level: median 5, IQR 2-6, U test p=0.23; duration: median 5, IQR 1-9, U test p=0.08). For the next observation period (2-4 min), however, the aggressiveness of the re-grouped crickets was significantly less than isolated crickets (n=57 interactions; median level 2, IQR 1-4, U test p=0.001; median duration 2s, IQR 0-5, U test p=0.001). At this time, as well as for the 3^rd^ and 4^th^ observation periods (4-6 and 6-8 min), neither the level nor the duration of agonistic interactions were significantly different to that recorded for crickets crowded since birth (U tests p> 0.05 in all cases). For the last observation period (8-10 min), the level and duration of interactions between re-grouped crickets tended to be less than that for the crowded-colony group (U tests: level p=0.0180 – not significant after Bonferroni alpha correction, duration p=0.0014), which could result from greater weight disparities between the contestants (cf. [49]), for which we could not control for in this experiment"

### Recovery from crowding and losing

Since each dyadic agonistic interaction always generates one submissive individual, we predict that the rapid decline in aggressiveness of isolated crickets on re-grouping is due alone to social subjugation, i.e. the loser effect. To add weight to this idea, we evaluated agonistic interactions between pairs of weight-matched, grouped crickets after being isolated for increasingly longer periods (3, 15, 30, 60 and 180 minutes), and compared this to the agonistic interactions between pairs of weight-matched losers, at corresponding times after defeat.

**Figure 4:**
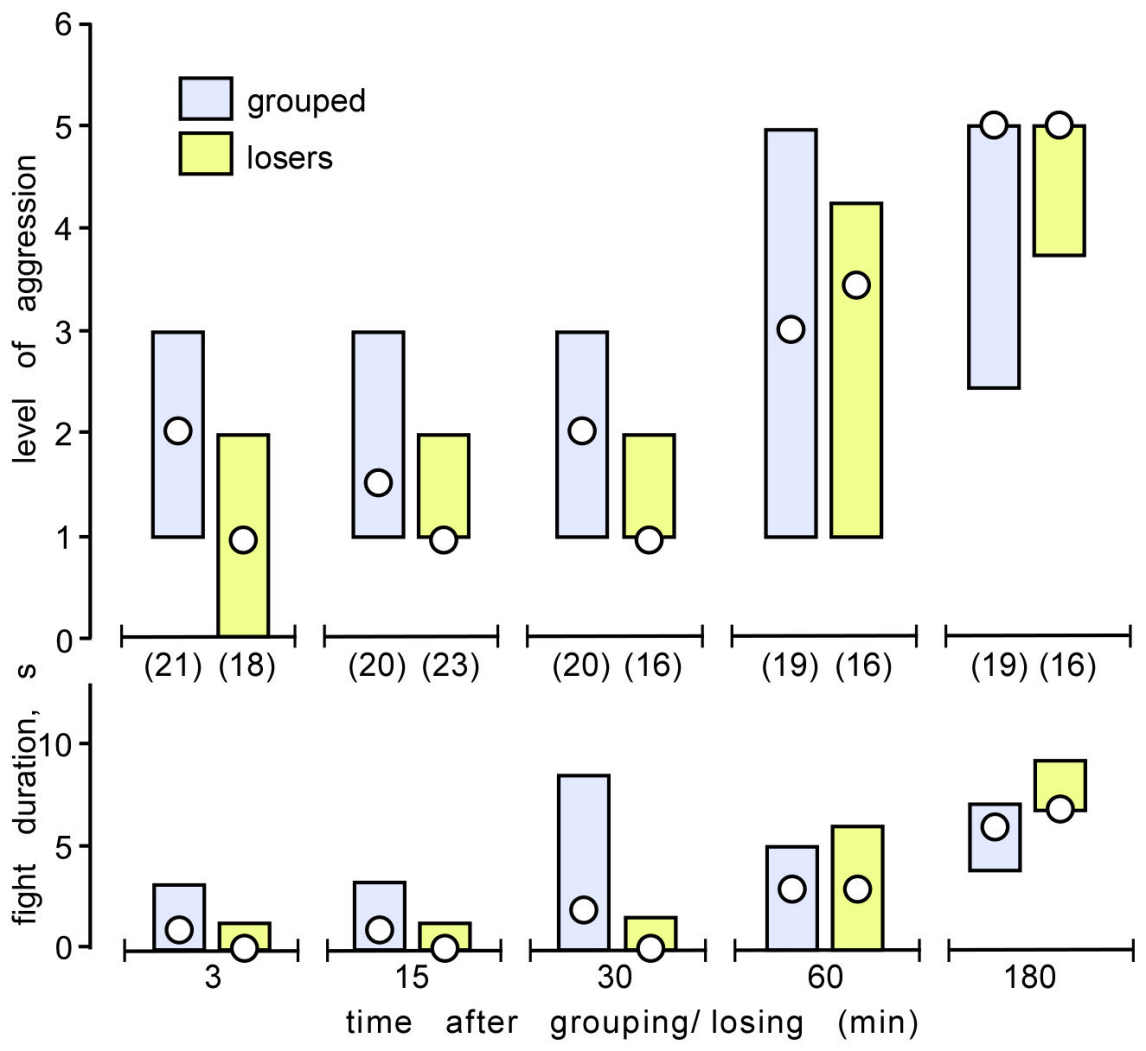
Recovery from crowding and losing. (A, B) Bar graphs comparing the level and duration respectively (circles: medians, bars: interquartile range) of aggression for encounters between pairs of male, weight matched crickets that were previously grouped (blue bars) or had lost a previous fight (losers; yellow bars). Each individual bar gives data from a different set of crickets each observed at different times after either isolation or losing; the number of pairs tested for each is given in parentheses under the x-axis in A. The differences between grouped and loser crickets for each time period are not statistically significant (Mann–Whitney U-test).

As shown in Figure 4, grouped crickets became progressively more aggressive over a period of 3 hours (Kruskal-Wallis Test: p-level=0.0067, p: duration < 0.001), after which time their aggressiveness was not significantly different to that exhibited by crickets isolated for at least one day (median level 5, IQR 2-5, U test p=0.15; median duration 7s, IQR 4-8, U test p=0.66).

## Discussion

The assertion in some quarters that social isolation increases an individual’s aggressiveness appears on perusing the literature to be a widely accepted dogma that stems perhaps from the misconception of viewing aggression as an aberrant behaviour in estranged individuals. Our work shows that in crickets at least, a turn of phrase is more appropriate: grouping lowers aggressiveness due to social subjugation, and social isolation allows a recovery to a default, naturally aggressive state.

Our account of the effects of isolation and crowding on aggression in male Mediterranean field crickets (Gryllus *bimaculatus* de Geer) substantiates earlier observations [10–12,50] that socially isolated individuals are far more aggressive than those maintained in groups. Whereas isolates invariably engage in physical fights lasting several seconds, grouped crickets rarely escalate beyond threat displays (Figure 1). This is also reflected in the probabilities of winning a contest. Grouped crickets are less likely than isolated crickets to defeat a near-standard, hyper-aggressive opponent (isolated-flown; Figure S1).

Contrary to free groups of crickets, crickets grouped together for a day in immediate proximity, but separated within individual mesh cages that permitted visual, olfactory and mechanosensory contact via the antennae, turned out to be as aggressive as completely isolated individuals (Figures 1 and S1). This was an unexpected result. Analogous experiments with fighting fish revealed that visual exposure to conspecifics significantly reduces aggression [51,52]. Furthermore, in the fruit fly Drosophila, evidence suggests that olfactory signalling alone could mediate the reduction of aggression in socially grouped males [53,54]. Similarly in locusts, a few short hours of olfactory and visual, or alone mechanosensory contact via the legs in *Schistocerca gregaria* [55], or antennae in *Chortoicetes* *terminifera* [56], is sufficient and necessary to change the social behaviour of solitarious individuals to that characteristic of the gregarious, swarm phase [4,57]. As in Drosophila [53,54,58] the pheromone signature perceived by the antennae in crickets is essential for species and sex recognition, and for inducing courtship and aggressive behaviours [50,59,60]), but not it seems for the subduing effect of grouping on aggression.

It could be argued that isolation in individual jars or cages counteracts any subduing influences of grouping since it represents a stressful situation [18], or establishes resident dominance by mimicking acquisition of a territory [20]. Although stressful conditions in crickets and other insects are known to lead to an increase in the release of octopamine [61], an amine that promotes the expression of aggression (reviews [35,36]:), the finding that the brains of isolated crickets contain less octopamine than crowded ones [62] suggests that isolation is unlikely to represent a stressful condition. Furthermore, while occupancy of a dark shelter leads in crickets to heightened aggressiveness due to activation of the octopaminergic system, occupancy of a small arena or a wire shelter was found to have no effect on aggression [40]. Hence, neither isolation within a glass jar nor caging would evoke a residency effect in our crickets.

It could nonetheless still be argued, that isolated males may be more aggressive since their situation suggests that females are scarce, and they hence fight more fiercely on confronting any conspecific male to ensure dominance and the securing of a female when encountered. Indeed, it has been reported that the presence of females "removes the effect of high population density on cricket aggression" [63]. Conversely, grouped males may be less aggressive since in the presence of numerous competitors the majority may switch to adopting a non-aggressive “satellite strategy”, waiting to intercept a female attracted to more dominant males [64]. The influence of females, and in particular copulation on cricket aggression is presently conjectural. While subordinate male *Acheta* *domesticus* that copulated with females are reported to be significantly more aggressive [65], (see also 10), findings in *Gryllus* *pennsylvanicus* suggest that mating is detrimental to success in aggressive contests [66] (see also 67 on *A* *. domesticus* ). Irrespective thereof, our experiments revealed no indication that the effect of isolation and crowding on aggression in *G* *. bimaculatus* results from the absence or presence of copulation partners. Firstly, grouped males housed together with ample virgin females were no more or less aggressive than males from groups without females. Secondly, single males isolated with a single virgin female were equally as aggressive as males isolated without sexual partners. While a female, as a key resource, can no doubt influence male aggression (our crickets for example tended to fight more aggressively in their presence) their absence can be neither the prime cause of hyper-aggressiveness in isolated males, nor subdued aggressiveness in grouped males.

The most parsimonious explanation for our findings is that low aggression in grouped crickets is a net consequence of social subjugation. Supporting this, isolated crickets, when grouped, initially engage in vigorous fights, but their intensity (level) and duration rapidly decline such that the average aggressiveness is as low as life-long crowded crickets within only 10 min (Figure 3). Although our crickets do not establish stable social hierarchies, this observation parallels that for individually housed animals as diverse as crayfish [68] and rhesus monkeys [69], which when grouped initially engage in extreme forms of aggressive behaviour that gradually declines as the group becomes socially organized by establishing a dominance hierarchy. For crickets, we suggest that the observed decline in aggression after initial grouping is a manifestation of the so-called loser effect. In nearly all species, aggressive interactions between conspecifics radically changes the contestants’ future behaviour such that winners (dominants) tend to become more aggressive and losers (subordinates) far less so [7]. Male crickets that have won a fight exhibit a relatively brief period of heightened aggressiveness that lasts under 20 min [40], whereas losers generally retreat on contacting any conspecific male [10], and require more than 1 and up to 24 hours to regain their aggressiveness [11,41,42]. Accordingly, due to aggressive encounters between grouped crickets, the majority of individuals at any given time greater than 10 min after crowding can be expected to be recovering from social subjugation, and hence the group would be on average far less aggressive than crickets isolated for a few hours. This suggestion is fully supported by our finding that grouped crickets acquire the aggressive status of isolates within only 3 hours of being isolated, on a time course exactly matching that required by isolates to recover their aggressiveness after losing a fight (losers, Figure 4).

We conclude that the lower levels of aggression exhibited by crowded crickets results from social subjugation, and that social isolation allows recovery to a state of heightened aggressiveness, which in crickets can be considered as the default condition. This conclusion is compatible with earlier proposals that heightened aggressiveness of isolates may be due to removal of social inhibition of aggression [23], forgetting prior agonistic experience [24], or a return to an individual aggressive level following a period of “social learning of non-aggressive behaviour" in isolation [70]. To our knowledge, however, we are the first to present clear evidence that the effects of crowding and isolation on animal aggression are manifestations of the loser effect and recovery there from.

Although we found no significant differences in aggression between crickets that were isolated for 1, 2 and 6 days, or between crickets grouped for only a few hours or lifelong, it cannot necessarily be inferred that social experiences have no long term consequence for the expression of aggression in crickets. Agonistic behaviour is known to enhance neurogenesis [71] and cFOS expression in the brain of male Acheta *domesticus* [72], while Gryllus integer males raised in the absence of the conspecific song are claimed to become more aggressive when adult [73]. It thus seems possible that long term changes in population density may have subtle influences on aggression that escape detection with the observation methods employed by us.

Given the widespread occurrence of the loser effect in the Animal Kingdom [7] our findings are relevant for interpreting the effects of isolation on aggression in all animals. In male rodents, for example, social defeat also inhibits competitive aggression [74], has similar physiological effects as social isolation and can lead to long lasting influences on the expression of adaptive social behaviour [75], changes in neuronal gene expression [76] and a depressive like state [77]. Furthermore, while long-term isolated mice show symptoms resembling those of depression and anxiety disorders [26], their aggressive behaviour has long been noted to be similar to that of dominants [78].

Isolation and crowding in insects can have wide-reaching physiological effects as dramatic as those occurring in vertebrates (reviews [1,3,5]:). For example, social isolation results in pronounced reduction in brain neuropil sizes in fruit flies [79], honey bees [80] and locusts [81] and is accompanied by fluctuations in the levels of different biogenic amines (crickets [12]:, locusts [31]:) and their receptors (Drosophila [15]: locusts [32]:). The primary causes and effects of population density associated changes in brain function on aggressive behaviour will, of course largely depend on the social structure of the animals in question. Migratory locusts, for example respond naturally to fluctuations in population density with striking, phenotypic plasticity, but do not exhibit pronounced territorial aggression, even when solitarious [4,33]. In eusocial insects, again in contrast to crickets, the tendency of an individual to fight members of a competitive group can actually increase with group size (e.g. [82,83]). We speculate that in non-social animals that exhibit territorial intraspecific aggression, such as crickets, the initial effects of social isolation will largely reflect recovery from social subjugation. In our opinion, this possibility has been neglected in the past.

## Supporting Information

Figure S1 **Effects of crowding and isolation on cricket aggression.** (A, B) Bar graphs giving the level and duration respectively (circles: medians, bars: interquartile range) of aggression for fights of selected test groups (as shown also in Fig. 1) against isolated males of corresponding weight that were flown to maximize their aggressiveness: grouped (20 per group, light blue bar), caged-grouped (light blue, hatched bar), caged-isolated (red, hatched bar), isolated for 1 day (red bar). (C) Gives the win frequencies of test group animals against flown isolates. Numbers in parentheses above the x-axis in A give the number of pairs or crickets for each group. Significant differences between groups are indicated (A, B: Mann–Whitney U-test, C: chi-square, * p < 0.05, ** p < 0.01, *** p < 0.001, ns not significant).
(TIF)
