# Supplementary figures and images for: Isolation Associated Aggression – A Consequence of Recovery from Defeat in a Territorial Animal
Source: PLoS One. 2013 Sep 6;8(9):e74965. doi: 10.1371/journal.pone.0074965 (PMC3765410; doi:10.1371/journal.pone.0074965)

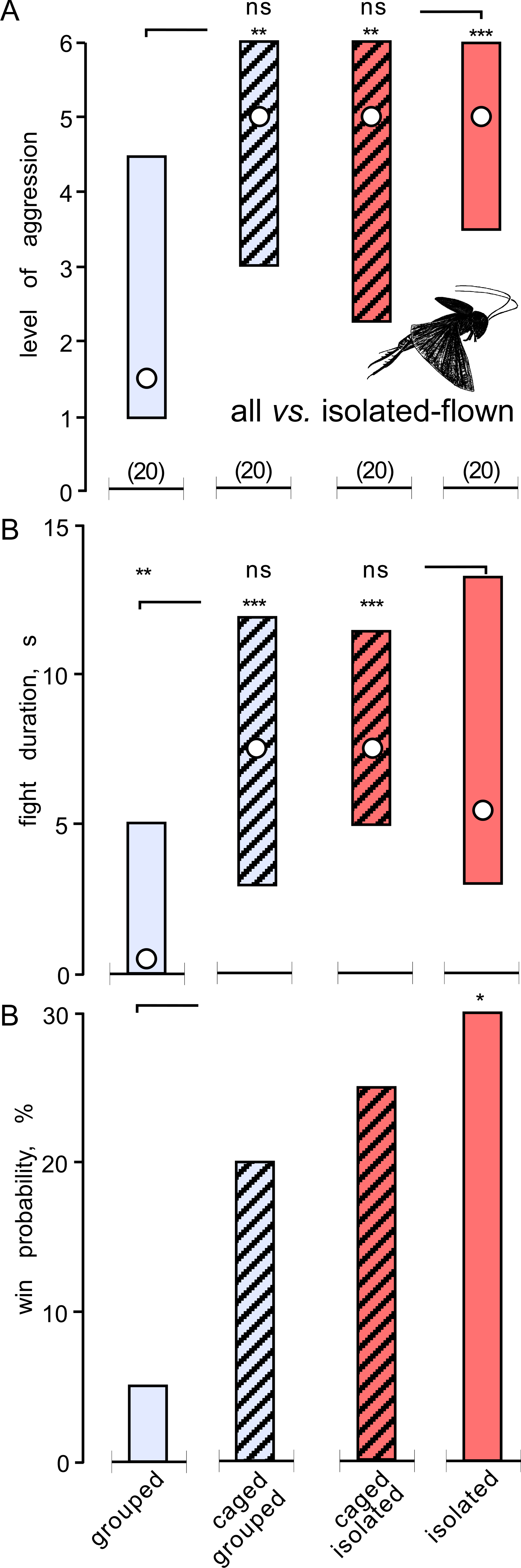

Supplement: Figure S1 — Effects of crowding and isolation on cricket aggression. (A, B) Bar graphs giving the level and duration respectively (circles: medians, bars: interquartile range) of aggression for fights of selected test groups (as shown also in Fig. 1) against isolated males of corresponding weight that were flown to maximize their aggressiveness: grouped (20 per group, light blue bar), caged-grouped (light blue, hatched bar), caged-isolated (red, hatched bar), isolated for 1 day (red bar). (C) Gives the win frequencies of test group animals against flown isolates. Numbers in parentheses above the x-axis in A give the number of pairs or crickets for each group. Significant differences between groups are indicated (A, B: Mann–Whitney U-test, C: chi-square, * p < 0.05, ** p < 0.01, *** p < 0.001, ns not significant). (TIF) [file pone.0074965.s001.tif]
